# The application of the One Health approach in the management of five major zoonotic diseases using the World Bank domains: A scoping review

**DOI:** 10.1016/j.onehlt.2024.100695

**Published:** 2024-02-15

**Authors:** Bir Doj Rai, Gizachew A. Tessema, Lin Fritschi, Gavin Pereira

**Affiliations:** a Curtin School of Population Health, Curtin University, 400 Kent St, Bentley, Perth, Western Australia 6102, Australia; b enAble Institute, Curtin University, 400 Kent St, Bentley, Perth, Western Australia 6102, Australia

**Keywords:** One Health, Zoonotic diseases, Physical infrastructure, Stakeholders, Financial and personal resources, Governance, Communication

## Abstract

The international authorities, such as the Food and Agriculture Organization of the United Nations, World Health Organization, World Organization for Animal Health, United Nations Environment Programme, and World Bank, have endorsed the One Health concept as an effective approach to optimize the health of people, animals, and the environment. The One Health concept is considered as an integrated and unifying approach with the objective of sustainably balancing and optimizing the health of people, animals, and ecosystems. Despite variations in its definitions, the underlying principle remains consistent – recognizing the interconnected and interdependent health of humans, animals, and the environment, necessitating interdisciplinary collaboration to optimize health outcomes. The One Health approach has been applied in numerous countries for detecting, managing, and controlling diseases. Moreover, the concept has found application in various areas, including antimicrobial resistance, food safety, and ecotoxicology, with a growing demand. There is a growing consensus that the One Health concept and the United Nations Sustainable Development Goals mutually reinforce each other. The World Bank has recommended five domains as foundational building blocks for operationalising the One Health approach, which includes: i) One Health stakeholders, roles, and responsibilities; ii) financial and personal resources; iii) communication and information; iv) technical infrastructure; and v) governance. The domains provide a generalised overview of the One Health concept and guide to its application. We conducted a scoping review following the five-staged Arksey and O’Malley's framework. The objective of the review was to map and synthesise available evidence of application of the One Health approach to five major zoonotic diseases using the World Bank domains. Publications from the year 2004, marking the inception of the term ‘One Health,’ to 2022 were included. Information was charted and categorised against the World Bank domains identified as *a priori*. We included 1132 records obtained from three databases: Embase, Medline, and Global Health; as well as other sources. After excluding duplicates, screening for titles and abstracts, and full text screening, 20 articles that contained descriptions of 29 studies that implemented the One Health approach were selected for the review. We found that included studies varied in the extent to which the five domains were utilised. Less than half the total studies (45%) used all the five domains and none of the studies used all the sub-domains. The environmental sector showed an underrepresentation in the application of the One Health approach to zoonotic diseases as 14 (48%) studies in 10 articles did not mention it as a stakeholder. Sixty two percent of the studies mentioned receiving support from international partners in implementing the One Health approach and 76% of the studies were supported by international donors to conduct the studies. The review identified disparate funding mechanisms employed in the implementation of the One Health approach. However, there were limited discussions on plans for continuity and viability of these funding mechanisms in the future.

## Highlights

• Evaluating the implementation of the One Health approach is required to assess their success and sustainability of such initiatives.
• While an increasing number of diverse stakeholders are engaged in implementing the One Health approach, the environmental sector, crucial for any public health intervention, is notably underrepresented.
• International partners play a vital role in the success of One Health initiatives by contributing essential financial and multi-sectoral human resources and engaging in exchange of knowledge and experiences, especially in low- and middle-income countries.

## 1 Introduction

The concept of One Health was officially launched in 2004 as ‘One World, One Health’ in a conference held by the Wildlife Conservation Society in the United States [1]. One Health is considered as an integrated, unifying approach that aims to sustainably balance and optimize the health of people, animals, and ecosystems [2]. Despite availability of various definitions of One Health concept [[2], [3], [4], [5], [6], [7], [8], [9]], (Supplementary file 1) its principle revolves around recognizing the interconnectedness of human, animal, and environmental health and using a collaborative, interdisciplinary approach to address complex health challenges at this intersection. The concept has been endorsed by major international authorities including the Food and Agriculture Organization of the United Nations (FAO), World Health Organization (WHO), World Organization for Animal Health (WOAH), World Bank, United States Agency for International Development (USAID) and Centres for Disease Control and Prevention (CDC) to promote effective, multisectoral and multidisciplinary collaboration [[10], [11], [12]]. A Quadripartite memorandum of understanding that was signed between FAO, WHO, WOAH, and United Nations Environment Programme (UNEP) in March 2022 offers a legal and formal framework to address the challenges at the human, animal, and ecosystem interface using an integrated and coordinated approach [13].

One Health approach has been implemented in many countries in the detection and control of zoonotic disease [[14], [15], [16], [17], [18], [19], [20]], with an increasing application in recent years in response to a surge in outbreaks [21,22]. Avian influenza, rabies, anthrax, and brucellosis are the priority zoonoses across multiple countries [[23], [24], [25], [26], [27], [28], [29], [30], [31], [32], [33], [34]] and they provide good cases to highlight the relevance of the One Health approach [[35], [36], [37]]. Scrub typhus represents a re-emerging vector-borne zoonosis [[38], [39], [40], [41], [42], [43], [44]], with close associations with environmental factors [[45], [46], [47], [48]]. Evidence of success from One Health approaches from around the globe and the increasing trend in emerging infectious disease outbreaks will enhance the utilisation of One Health in the future.

There is a growing consensus on the mutually reinforcing relationship between the One Health concept and the United Nations Sustainable Development Goals (SDGs) [49]. The One Health approach facilitates meeting the SDGs [50]. Six of the SDGs directly address the One Health approach: SDG3 (Good Health and Wellbeing); SDG6 (Clean water and sanitation, SDG11 (Sustainable cities and communities); SDG13 (Climate action); SDG14 (Life below water); and SDG15 (Life on land) [51]. Most SDGs are interconnected and requires a paradigm based on inter-sectoral integration and collaboration, thereby providing a unique opportunity for One Health approaches [52].

The World Bank has provided an operational framework [8] to allow operationalisation of One Health both for target countries and the donors [53,54]. The five domains in the framework are the foundational building blocks of One Health [8], comparable to the health building blocks of the World Health Organization [55,56] and with adaptability to be employed across diverse contexts [8,56]. These domains are: i) One Health stakeholders, roles, and responsibilities; ii) financial and personal resources; iii) communication and information; iv) technical infrastructure; and v) governance.

Many studies have highlighted the application of One Health approaches to specific diseases or a program [17,[57], [58], [59], [60]]. However, there apprears to be inconsistencies and disparities in fully implementing the domains of One Health, which will impact the effectivess of the approach.

To date, no systematic or scoping review has been conducted to map and synthesise available evidence to highlight best practice or explore gaps in the implementation of One Health using the World Bank domains. The aim of this scoping review was to map and synthesise available evidence describing the implementation of One Health approach against five major zoonotic diseases globally using the World Bank domains.

## 2 Methods

We followed the Joanna Briggs Institute (JBI) Manual for Evidence Synthesis [61] which was built from the original framework of Arksey and O'Malley [62] later enhanced by Levac et al. [63] The framework consists of five sequential stages: identifying the research question, identifying relevant studies, study selection, charting the data and collating, summarising, and reporting results. Optional stage six, ‘consultation,’ was not conducted as validation of the results are not necessary in the context of a scoping review. The Preferred Reporting Items for Systematic Reviews and Meta-Analyses Extension for Scoping Reviews (PRISMA-ScR) [64] checklist was used for reporting the review (Supplementary file 2).

### 2.1 Objectives and scope

The aim of this scoping review was to map and synthesise available evidence describing the application of the One Health approach against five major zoonotic diseases globally using the World Bank domains.

### 2.2 Identification of relevant studies

We used the population, concept, and context (PCC) framework from the JBI Manual for Evidence Synthesis [61] to develop inclusion and exclusion criteria (Supplementary file 3). Population: The population included all animal, human and environment population to which the One Health approach was implemented. Concept: Operationalisation of One Health approach with application of one or more of the World Bank domains to at least one of anthrax, brucellosis, avian influenza, rabies, and scrub typhus [[35], [36], [37]]. Context: Studies from any geographic location were included whereby the intention of the study was interpreted as to inform surveillance, response, control of zoonotic diseases.

A three-step search process was conducted. First, an initial search was conducted on the Global Health database to identify index terms, medical subject headings (MeSH) and synonyms of the diseases in titles and abstracts. As the objective of the search was to retrieve papers describing the implementation of the One Health approach, the term “One Health” in combination with the selected zoonotic diseases was identified as one of the key index terms in the search strategy. In the second step, a search using all key concept terms and disease synonyms was undertaken across Embase, Medline and Global Health because of their wide scope of publications and multidisciplinary contents [65]. Boolean, truncations, and wild cards were used in various combinations with these keywords and index terms. A librarian from the Faculty of Health Sciences, Curtin University was consulted for designing the search strategy (Supplementary file 4). Third, a search was conducted using the Google search engine and Google Scholar for non-indexed studies on webpages of WHO, WOAH, FAO, and One Health Commission and reference lists of studies initially retrieved.

### 2.3 Study selection

A total of 1132 records were identified, of which 1117 records were identified through the database search and 15 records through the search on web pages and from references of initially selected papers. After removing 602 duplicate records, 530 eligible records were selected for title and abstract screening. GP, GAT and LF screened 30 papers with queries regarding selection, and the rest of the papers were screened by BDR. We removed 420 records after title and abstract screening leaving 110 records for full text screening. Ninety records not meeting the inclusion criteria were removed after full text screening leaving 20 records for analysis. The results of screening process were recorded using the PRISMA flow diagram.

### 2.4 Data charting

Distinct components, equivalent to sub-domains, were identified under each domain based on findings from other relevant studies (Table 1). This approach was employed to ensure a clear and focused assessment of the literature under each domain. The information charted were standard bibliographic information that included the author(s), year of publication, aims and purpose of the studies, countries where the studies were conducted, diseases against which One Health was operationalised, and any information encompassing domains and subdomains (Supplementary file 5). An iterative process of screening and extracting data was undertaken, discussed, and reviewed by co-authors. In cases of articles that were inconclusive, research team members discussed to reach a decision.

**Table 1:** Domains and subdomains with their sources.

Domains⁎	Desired components (Sub-domains)	Adapted from
1. Stakeholders, roles, and responsibilities	• Identification of stakeholders. • Stakeholders from animal, human and environment sectors. • Stakeholders beyond animal, human and environment sectors. • International stakeholders. • Roles of stakeholders at various levels.	Fasina et al., 2021 [99], Vesterinen et al., 2019 [116].
2.Financial and personal resources	• Funding mechanisms. • Sustainability of fundings. • Funding sources. • Capacity building programs.	Fasina et al., 2021 [99], Ruegg et al., 2018 [95].
3. Communication and information	• Channels for information sharing- Meetings and forums. • Agreed communication strategy among stakeholders. • Use of any real-time data sharing methods.	Vesterinen et al., 2019 [116], Mahrous et al., 2020 [117], Lokossou et al., 2021 [118].
4. Technical infrastructure	• Health system set-up. • Laboratories set-up and their competencies. • Laboratories and staff competencies.	Paternoster et al., 2017 [57], Macedo Couto et al., 2020 [119].
5. Governance	• National policy on OH • Government bodies, and intersectoral agreements dedicated to OH. • Government laws and policies implicating OH approaches.	Mahrous et al., 2020 [117], Lokossou et al., 2021 [118] , Couto et al., 2020 [119].

⁎ Domains extracted from: The World Bank. *Operational Framework for Strengthening Human, Animal, and Environmental Public Health Systems at their Interface*. 2018 [8].

### 2.5 Collating, summarising, and reporting results

The extracted information from the included articles were presented in a tabular form and synthesised. Articles that contained more than one One Health operationalisation study or One Health operationalisation to more than one zoonoses were identified and enumerated. Information from individual studies described in each article were tabulated and organised along the World Bank domains and subdomains identified *a priori.* Frequency counts of the articles, individual studies described in each article, geographical regions, zoonotic diseases to which the One Health approach was utilised, and types and frequencies of discussions on World Bank domains and sub-domains were tabulated and analysed.

## 3 Results

### 3.1 Characteristics of selected studies

**Fig. 1:**
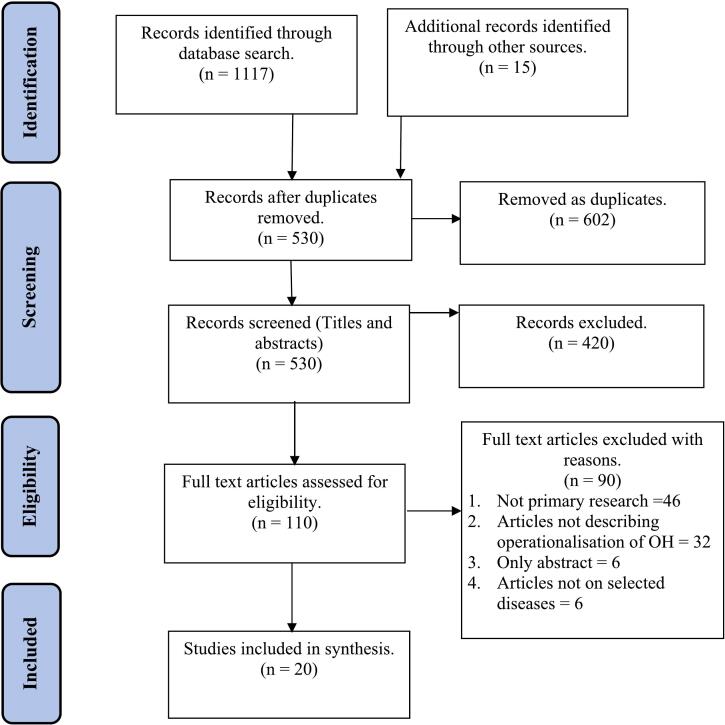
Selection process in PRISMA flow diagram. Adapted from: Moher D, Liberati A, Tetzlaff J, Altman DG, Group P. Preferred reporting items for systematic reviews and meta-analyses: the PRISMA statement. *PLoS Med*. 2009;6(7):e1000097. doi:10.1371/journal.pmed.1000097 [115]

After removing duplicates and screening we included 20 articles in the final review (Fig. 1). The included articles contained 29 studies describing the One Health implementation from 15 countries (Table 2). Some articles contained multiple studies, with three articles describing studies in more than one disease [34,60,66] and four articles describing it in more than one country [60,[67], [68], [69]]. Seven articles provided evidence of 14 (48%) One Health studies from the sub-Saharan Africa [34,60,66,68,[70], [71], [72]] followed by five articles describing six studies (21%) in the East Asia & Pacific region [69,[73], [74], [75], [76]].

**Table 2:** Characteristics of the selected studies.

Author(s) and year	Study aims	Countries	Zoonoses
Abbas et al., 2011 [79]	To review the activities against rabies and effect of intersectoral co-ordination on dogs' bites and rabies cases.	India	Rabies
Masthi et al., 2014 [78]	To evaluate efficacy of rabies vaccines and demonstrate benefits of One Health against human rabies and animal bite cases.	India	Rabies
Okello et al., 2014 [60]	To study the emerging relationships between international One Health dialogue and its practical implementation in the African health policy.	Tanzania	Rabies
		Nigeria	Avian Influenza
Mpolya et al., 2017 [71]	To describe a demonstration project for elimination of rabies.	Tanzania	Rabies
Mwakapeje et al., 2017 [72]	To describe implementation of One Health approach against anthrax.	Tanzania	Anthrax
Rock et al., 2017 [77]	To assess policies for dog-bites and rabies.	Canada	Rabies
Barroga et al., 2018 [76]	To describe the development of a One Health frame- work in a multisectoral approach.	Philippines	Rabies
Buttigieg et al., 2018 [67]	To demonstrate and compare use of One Health approach to brucellosis in Malta and Serbia.	Malta	Brucellosis
		Serbia	Brucellosis
Hermesh et al., 2019 [82]	To address the benefits of expanding the One Health perspective to include ethical and the socio-political aspects of health.	Israel	Brucellosis
Hort et al., 2019 [69]	To draw lessons on values of stewardship of stakeholders against infectious diseases to enable adoption of One Health approaches.	Thailand	Avian Influenza
		Indonesia	Avian Influenza
Nihal et al., 2019 [80]	To review surveillance data and assess need and potential of One Health approach for surveillance of rabies.	Sri Lanka	Rabies
Standley et al., 2019 [34]	To define and prioritise the zoonotic diseases. To identify gaps and challenges in zoonotic disease prevention and control procedures.	Republic of Guinea	Rabies
			Anthrax
			Brucellosis
			Avian Influenza
Lushasi et al., 2020 [70]	To assess feasibility and possible impact of Integrated Bite Case Management for rabies surveillance in Tanzania.	Tanzania	Rabies
Yasobant et al., 2020 [83]	To identify and categorize stakeholders for control and prevention of zoonotic diseases at the human–animal health system interface and identify challenges in intersectoral collaboration during outbreak and peace time.	India	Avian Influenza (zoonoses as general)
Zuhriyah et al., 2020 [75]	To identify the factors required for control of the rabies in Domphu, Indonesia.	Indonesia	Rabies
Dargantes et al., 2021 [74]	To assess the integration of Rabies management information system into the rabies prevention and control program through One Health approach in the Philippines.	Philippines	Rabies
Lechenne et al., 2021 [68]	To describe One Health research project and evaluate its success.	Chad	Rabies
		Côte d'Ivoire	Rabies
		Mali	Rabies
Mansingh et al., 2021 [81]	To identify gaps in controlling the animal and human anthrax cases.	India	Anthrax
Innes et al., 2022 [73]	To describe and evaluate One Health pilot program for surveillance of Avian influenza.	Thailand	Avian Influenza
John et al., 2022 [66]	To assess preparedness and response to public health issues (Anthrax and rabies) at various levels.	Tanzania	Rabies
			Anthrax

Rabies emerged as the most frequently investigated zoonotic disease, accounting for 52% of the total studies described in 13 articles [34,60,66,68,70,71,[74], [75], [76], [77], [78], [79], [80]]. Description of the One Health implementation against scrub typhus was not found in any of the selected articles.

The reviewed studies mentioned at least one of the five domains of One Health. 16 (45%) mentioned all the domains while none of the studies mentioned all the sub-domains. While stakeholders were mentioned in all the studies in the selected articles, technical infrastructure was described the least (18 studies, 62%) in 12 articles [34,66,[68], [69], [70], [71], [72], [73],^75^,[80], [81], [82]]. Evidence on application of One Health and its domains was greater in Low- and Middle-Income Countries (LMICs) with 23 (79%) studies in 15 articles from LMICs [34,60,66,68,[70], [71], [72],[74], [75], [76],[78], [79], [80], [81],^83^]. Of all the LMICs the majority of studies were from Tanzania (20%) and India (14%).

### 3.2 Implementation of One Health in Zoonotic diseases

#### 3.2.1 Stakeholders, roles, and responsibilities

In the review, all the selected articles described a range of One Health stakeholders which included community members at the lowest level to international organisations at the highest level (Table 3). Stakeholders described were either government institutions, non-government organisations or private parties with varying backgrounds. Private sector vaccine manufacturers, private clinicians/physicians/veterinarians, private poultry corporation were mentioned by three articles [68,73,83]. All studies mentioned stakeholders from animal and human health sectors, and 14 (48%) studies in 10 articles did not mention the environmental sector as a stakeholder [60,67,68,70,74,[77], [78], [79],^82^]. Apart from animal, human and environmental health sectors, other stakeholders included finance, regulatory, academic and research, government administrative units, social sectors, and volunteers and were stated by 15 articles in 21 (72%) studies [ 60,[66], [67], [68], [69],^71^,^72^,[74], [75], [76],[78], [79], [80], [81],^83^]. Thirty four percent of studies contained in six articles mentioned international organisations as stakeholders [66,68,69,71,73,75]. The roles played by national stakeholders were based on their institutional mandates. For example, stakeholders from animal health sectors, human health sectors and administrative units were involved in surveillance of animal populations, human populations and providing administrative support, respectively. The international organisations main roles were to provide technical and financial support.

**Table 3:** Extracted information along the domains and their sub-domains.

Author(s) and year	Country, zoonoses	Domain: Stakeholders, roles, and responsibilities	Domain: Financial and personal resources	Domain: Communication and information	Domain: Technical infrastructure	Domain: Governance
		**Sub-domains:**# 1. Stakeholders from the animal health, human health, and environmental (AHE) sectors. 2. Representation from the AHE sectors. 3. Stakeholders beyond AHE sectors. 4. Private sectors stakeholders. 5. International stakeholders.	**Sub-domains:**# 1. Funding source and mechanism. 2. External/international support. 3. Sustainability of funding. 4. Staff capacity as challenge. 5. Staff insufficiency as challenge. 6. Mention on trainings. 7. Study funding.act	**Sub-domains:**# 1. Information sharing mechanism. 2. Electronic systems for real-time information sharing. 3. Awareness.	**Sub-domains:**# 1. Health facilities names and set up. 2. Laboratory names and set up. 3. Laboratory capacities/deficiencies. 4. Laboratory staff capacity.	**Sub-domains:**# 1. National OH plans/strategies. 2. Government's commitment/will. 3. OH platform for intersectoral collaboration. 4. Government bylaws and policies.
**Abbas et al., 2011** [79]	India, rabies.	1. Directorates of Public Health & Preventive Medicine, Medical Education, Rural Health & Medical Services, State Surveillance Office and Medical Services Corporation, animal welfare department, Department of Animal Husbandry. 2. Animal and human. 3. Municipal administration department. 4. Civil society organisations, local animal welfare organization. 5. None.	1 to 5. None. 6. Training was given to human health sectors on ID vaccine administration. 7. WHO.	1 and 2 None. 3. Awareness campaigns conducted on rabies and animal rights.	1 to 4. None.	1. None. 2. Demonstration of a political commitment from the government. 3. District level monitoring committee and state level coordination committees. 4. Licensing of dog rules helped in rabies control.
**Masthi et al., 2014** [78]	India, rabies.	1. Medical and veterinary professional. 2. Animal and human. 3Village leaders, self-help groups, animal welfare professionals, schoolteachers, and village level volunteers. 4 and 5. None.	1 to 5. None. 6. Training was conducted for animal and human health sectors and other stakeholders. 7. Global Alliance for Rabies Control.	1 to 3. None.	1 to 4. None.	1 to 4. None.
**Okello et al.,** 2014 [60]	Tanzania, rabies.	1. Animal Health and human health sectors. 2. Animal and human. 3. National academic and research institutes. 4 and 5. None.	1. National budget used alongside donor funding. 2. Bill and Melinda Gates Foundation. 3 and 4. None. 5. Insufficient staff in the field 6 and 7. None.	1 to 3. None.	1 to 4. None.	1 to 4. None.
	Nigeria, avian influenza.	1. Animal Health and human health sectors. 2. Animal and human. 3. National academic and research institutes. 4 and 5. None.	1. None 2. World Bank. 3 to 5. None 6. National Field Epidemiology and Laboratory Training Programme conducted. 7. European Union.	1 to 3. None.	1 to 4. None.	1. None. 2. Unprecedented political and financial backing. 3. The National Technical Committee on Avian Influenza. 4. None.
**Mpolya et al.,** 2017 [71]	Tanzania, rabies	1. The Ministry of Health, Community Development, Gender, Elderly and Children, the Ministry of Agriculture, Livestock and Fisheries. 2. Animal and human. 3. Research and academic institutes. 4. None. 5. WHO.	1. Multiple sources of national funding. 2. Bill and Melinda Gates Foundation, the Wellcome Trust, the UBS Optimus Foundation. 3 to 5. None. 6. Training was provided to animal and human health sectors. 7. Bill and Melinda Gates Foundation, the Wellcome Trust, the UBS Optimus Foundation, the Medical Research Council and the Science and Technology Directorate, Department of Homeland Security, Fogarty International Centre, and the National Institute of Health.	1. None. 2. Established a mobile phone-based surveillance system. 3. Awareness programs conducted on rabies.	1. None. 2. The Tanzania Veterinary Laboratory Agency and the Sokoine University of Agriculture's, Faculty of Veterinary Medicine Laboratory in Morogoro. 3. Logistical challenges in sample handling and submission. 4. None.	1 and 2. None. 3. One Health Coordination Unit. 4. None.
**Mwakapeje et al.,** 2017 [72]	Tanzania, anthrax.	1. Ministry of Health, Community Development, Gender, Elderly and Children, regional and districts medical/veterinary offices, game office. 2. Animal, human and environment. 3. Experts from the Prime Minister's Office Research Institute. 4 and 5. None.	1 to 4. None. 5. Shortage of staff in animal and human health sectors. 6 and 7. None.	1. None. 2. Electronic Integrated Disease Surveillance and Response System. 3. Awareness programs conducted on rabies.	1. None. 2. The Tanzania Veterinary Laboratories Agency. 3. None. 4. Shortage of experts.	1. National One Health Strategic Plan for the period 2015–2020. 2. None. 3. The One Health Coordination Unit. 4. None.
**Rock et al.,** 2017 [77]	Canada, rabies.	1. Physicians specializing in public health and preventive medicine, animal control officer, supervisor, and veterinarians. 2. Animal and human. 3 to 5. None.	1. Reinvestment of revenue generated into dog control activities. 2 to 6. None. 7. Canadian Institute of Health Research.	1 and 2. None. 3. Awareness on dog bites recommended.	1 to 4. None.	1 to 3. None. 4. Implementation of responsible pet ownership bylaw enabled coordination.
**Barroga et al.,** 2018 [76]	Philippines, rabies.	1. Municipal Veterinary Office, Departments of Health and Environment and Natural Resources. 2. Animal, human and environment. 3. Municipal Agriculture Office, local government units. 4 and 5. None.	1. None. 2. OIE and Australia's Department of Foreign Affairs and Trade. 3 and 4. None. 5. Shortage of staff in animal and human health sectors. 6. None. 7. Australia's Department of Foreign Affairs and Trade.	1 and 2. None. 3. Conducted awareness on rabies in school and community.	1 to 4. None.	1 and 2. None. 3. Philippine Inter-Agency Committee on Zoonoses. 4. None.
**Buttigieg et al.,** 2018 [67]	Malta, brucellosis.	1. Departments of Public and Environmental Health, and Agriculture and Veterinary Services. 2. Animal, human and environment. 3. Department of Consumer Affairs, Justice, and Police. 4 and 5. None.	1 to 6. None. 7. European Cooperation in Science and Technology.	1. Formal and informal sharing of information. 2. None. 3. Lack of awareness on brucellosis.	1 to 4. None.	1 to 3. None. 4. Strict enforcement of legislation helped in control of brucellosis.
	Serbia, brucellosis.	1. Directorate for Veterinary Services, Public Health. 2. Animal and human. 3. Internal Affairs Ministry 4 and 5. None.	1 to 6. None. 7. European Cooperation in Science and Technology.	1. Informal sharing of information. 2. None. 3. Lack of awareness on brucellosis.	1 to 4. None.	1 to 3. None. 4. Strict enforcement of legislation helped in control of brucellosis.
**Hermesh et al.,** 2019 [82]	Israel, brucellosis.	1. Human and animal health practitioners. 2. Animal and human. 3 to 5. None.	1 to 7. None.	1 to 3. None.	1. Negev's main hospital. 2 to 4. None.	1 to 3. None. 4. Veterinary regulations enforcement encountered lack of cooperation.
**Hort et al.,** 2019 [69]	Thailand, avian influenza.	1. Ministries of Public Health, Cooperatives and Natural Resource and Environment. 2. Animal, human and environment. 3. Universities, Ministries of Interior and Defence, local administration, and civil society. 4. Food and animal production sector. 5. WHO, USAID, CDC, FAO	1. Individual national agencies allocated their own budget. 2. FAO, WHO, USAID and USCDC. 3 to 7. None.	1 and 2. None. 3. Plans of public awareness programs and campaigns on EIDs in Thailand.	1 to 4. None.	1 and 2. None. 3. Thai One Health Network. 4. None.
	Indonesia, avian influenza.	1,Ministries of Health and Agriculture. 2. Animal and human. 3. In Indonesia- Coordinating Ministry for Human Development and Cultural Affairs and the National Agency for Disaster Management, INDOHUN (government technocrats, practitioners, academics. 4. None. 5. FAO, OIE, WHO.	1. Funding through national disaster management agency. 2. USAID supported in pilot districts. 3 to 5. None 6. INDOHUN had conducted nine preservice and six in-service training programmes. 7. None.	1 to 3. None.	1. Provincial, district, subdistrict health centres. 2 to 4. None.	1 and 2. None 3. Indonesia One Health University Network (INDOHUN. 4. Law 41 of 2014 on Livestock and Animal Health Law 23/2014, responsibility for the detection and response to outbreaks of infectious disease were relevant to OH.
**Nihal et al., 2019** [80]	Sri Lanka, rabies.	1. Public health veterinary service, Department of animal production and health, Department of Wildlife, and conservation. 2. Animal, human and environment. 3. Research institute 4 and 5. None.	1. None. 2. International Development Research Committee of Canada. 3. None. 4. Absence of skilled staff in wildlife. 5. Lack of veterinarians trained in wildlife diseases 6. None. 7. International Development Research Committee of Canada.	1 to 3. None.	1. None. 2. Medical Research Institute central laboratory, peripheral laboratories in University of Peradeniya and Teaching Hospital, Karapitiya Laboratories. 3. Lack of infrastructure to collect and submit samples in wildlife. 4. None.	1 to 4. None.
**Standley et al.,** 2019 [34]	Republic of Guinea, rabies.	1. Ministries of Health, Environment and Livestock. 2. Animal, human and environment. 3 to 5. None.	1. None. 2. Donors and international partners. 3. None. 4. Lack expertise in animal and environment sector. 5. Limited human resources allocation. 6. Need for training at different levels. 7. USCDC.	1 and 2. None. 3. Lack of public awareness on One Health concept.	1. Health/ Livestock posts. 2. Central Veterinary Diagnostic Laboratory and Pasteur Institute of Dakar. 3. Limited laboratory capacity. 4. None.	1. One Health preparedness and response plan. 2 and 3. None. 4. No standard policy for collaboration for rabies at perfectual level. No harmonised policy and procedure for priority diseases.
	Republic of Guinea, anthrax	1. Ministry of Health, Ministry of Environment and Ministry of Livestock. 2. Animal, human and environment. 3 to 5. None.	1. None. 2. Donors and international partners. 3. None 4. Lack expertise in animal and environment sector. 5. Limited human resources allocation. 6. Need for training at different levels. 7. USCDC.	1 and 2. None. 3. Lack of public awareness on One Health concept.	1. Health/ Livestock posts. 2. Central Veterinary Diagnostic Laboratory. 3. Limited laboratory capacity. 4. None.	1. One Health preparedness and response plan. 2 and 3. None. 4. No harmonised policy and procedure for priority diseases.
	Republic of Guinea, brucellosis.	1. Ministry of Health, Ministry of Environment and Ministry of Livestock. 2. Animal, human and environment. 3 to 5. None.	1. None. 2. Donors and international partners. 3. None. 4. Lack expertise in animal and environment sector. 5. Limited human resources allocation. 6. Need for training at different levels. 7. USCDC.	1 and 2. None 3. Lack of public awareness on One Health concept.	1. Health/ Livestock posts. 2. Central Veterinary Diagnostic Laboratory and Pasteur Institute of Dakar. Regional labs 3. Limited laboratory capacity. 4. None.	1. One Health preparedness and response plan. 2 and 3. None 4. No harmonised policy and procedure for priority diseases.
	Republic of Guinea, avian influenza.	1. Ministry of Health, Ministry of Environment and Ministry of Livestock. 2. Animal, human and environment. 3 to 5. None.	1. None. 2. Donors and international partners. 3. Lack of sustained funding was a challenge. 4. Lack expertise in animal and environment sector. 5. Limited human resources. 6. Need for training at different levels. 7. USCDC.	1 and 2. None. 3. Lack of public awareness on One Health concept.	1. Health/ Livestock posts. 2. Central Veterinary Diagnostic Laboratory. 3. Limited laboratory capacity. 4. None.	1. One Health preparedness and response plan. 2. None. 3. Guinea's One Health Platform. 4. No standard policy for collaboration for rabies at perfectual level. No harmonised policy and procedure for priority diseases.
**Lushasi et al.,** 2020 [70]	Tanzania, rabies.	1. Ministries of Health and Livestock and Fisheries. 2. Animal and human. 3 to 5. None.	1 to 5. None. 6. Both health and animal sector staff were trained. 7. Wellcome Trust, the DELTAS Africa Initiative.	1. None. 2. Realtime Integrated Bite Case Management application. 3. None.	1. District hospitals. 2. The Tanzania Veterinary Laboratories Agency. 3 and 4. None.	1 and 2. None. 3. One Health Coordination Desk. 4. None.
**Yasobant et al.,** 2020 [83]	India, Avian Influenza as a case. (zoonoses as general)	1. Administrators of human health, animal health and environment sector 2. Animal, human and environment. 3. Community health workers, research institutes, and police. 4. Private clinicians/physicians, private veterinarians, NGOs, dairy farms, media, community leaders. 5. None.	1 to 4. None. 5. Shortage of human resources in animal sector. 6. None. 7. Ministry of Culture and Science of North Rhine-Westphalia, Germany.	1 and 2. None. 3.Lack of awaren ess among stakeholders and community on zoonoses.	1 to 4. None.	1. None. 2. Lack of political commitment was a challenge. 3 and 4. None.
**Zuhriyah et al.,** 2020 [75]	Indonesia, rabies.	1. Government of Domphu district, Directorate General of Livestock and Animal Health Services, human health institutes, and One Health Center,Indonesia One Health University Network. 2. Animal, human and environment. 3. Universities. 4. None. 5. FAO.	1. None 2. FAO provided training. 3 to 5. None. 6. Both health and animal sector were trained. 7. None.	1 and 2. None. 3. Low community awareness on OH concept.	1 and 2. None. 3. Inadequate technical capabilities and reduced laboratory infrastructure. 4. None.	1 to 3. None. 4. Lack of the regulatory capacity of the government as a challenge.
**Dargantes et al.,** 2021 [74]	Philippines, rabies.	1. Personnel from veterinary, public health. 2. Animal and human. 3. Social services and IT sectors. 4 and 5. None.	1. Reinvestment of revenue (Rabies Trust Fund). 2. None. 3. Sustainability feature incorporated in the strategy. 4. None. 5. Lack of manpower both in veterinary and human health sectors. 6. None. 7. GREASE (Gestion des Risques Emergents en Asie du Sud-Est) and the French Agricultural Research Centre for International Development.	1 and 2. None. 3. Lack of public awareness on OH concept.	1 to 4. None.	1 to 3. None. 4. The passage of Provincial Ordinance 412 in 2016, or the Revised Dog and Rabies Control Ordinance of the Province of Agusan del Norte, provided a legal framework in its implementation of OH and RabMIS.
**Lechenne et al.,** 2021 [68]	Chad, rabies.	1. Ministries of livestock and Health. 2. Animal and human. 3. Research and academic sectors. 4. Private sector vaccine manufacturers. a local Chadian Nongovernmental Organization. 5. Swiss TPH (WHO, PARACON, IPP-As a result of OH).	1. None. 2. Vaccine Alliance 3 to 5. None. 6. Trainings conducted. 7. Vaccine Alliance.	1. Meetings. 2. None. 3. Public awareness on rabies built up.	1. None. 2. laboratory in N‘Djamena. 3 and 4. None.	1 to 4. None.
	Côte d'Ivoire, rabies.	1. Ministries of Public Health and Agriculture. 2. Animal and human. 3. Research and academic sectors. 4. Private sector vaccine manufacturers. 5. Swiss TPH (WHO, PARACON, IPP-As a result of OH).	1. None. 2. Vaccine Alliance. 3 to 5. None. 6. Trainings conducted. 7. Vaccine Alliance.	1. Meetings. 2. None. 3. Public awareness on rabies built up.	1. Human anti-rabies centres and animal anti-rabies centres. 2. Central Pathology Laboratory at Bingerville and the Institut Pasteur of Cˆote d'Ivoire. 3 and 4. None.	1 and 2. None 3. Stakeholder platforms. 4. None.
	Mali, rabies	1. “Laboratoire Centrale V´et'erinaire”, Faculty of Medicine and Odontostomatology of the University of Sciences, Technics and Technologies of Bamako. 2. Animal and human. 3. Research and academic sectors. 4. Private sector vaccine manufacturers. 5. Swiss TPH (WHO, PARACON, IPP-As a result of OH).	1. None. 2. Vaccine Alliance. 3 to 5. None. 6. Trainings conducted. 7. Vaccine Alliance.	1. Meetings. 2. None. 3. Public awareness on rabies built up.	1. Community health centers and health reference centers. 2. “Laboratoire Rodolph M'erieux” in Bamako, and the central veterinary laboratory. 3 and 4. None.	1 and 2. None. 3. Stakeholder platforms. 4. None.
**Mansingh et al.,** 2021 [81]	India, anthrax.	1. Health Department, Veterinary Department, Forest Department. 2. Animal, human and environment. 3. Education department, Panchayati raj institutions, tribal welfare department, community. 4 and 5. None.	1 to 4. None. 5. Inadequate manpower in human and animal sector. 6. Training need- No guidance and training of lab personal in human health sector. 7. Indian Council of Medical Research.	1 and 2. None. 3. Lack of awareness among community on Anthrax.	1. None. 2. State level laboratories. 3. Anthrax diagnostic facility not available at the district level. Lack of laboratory support at the regional level. 4. No designated laboratory staff for diagnosis of anthrax.	1 to 4. None.
**Innes et al.,** 2022 [73]	Thailand, avian influenza.	1.Departments of Disease Control, Livestock Development, National Wildlife Parks and Plant Conservation. 2. Animal, human and environment. 3. None. 4. Private poultry corporation. 5. USCDC.	1. None 2. WHO, FAO, USCDC provided technical and funding assistance. 3. Sustainability mentioned as a recommendation. 4. None 5. Weak laboratory human resources. 6. Integrated trainings were provided to both animal and human sector. 7. Johns Hopkins Alliance for a Healthier World.	1. Meetings, informal and formal sharing of information. 2. Three mobile applications. 3. None.	1. None. 2. Regional laboratories network and central laboratories. 3. Limited human resources. 4. None.	1. None. 2. Lack of political will reported. 3. Coordinating Unit for One Health. 4. None.
**John et al.,** 2022 [66]	Tanzania, rabies.	1. Ministries of Livestock and Fisheries, Health, Community Development, Gender, Elderly and Children and Natural Resources and Tourism. 2. Animal, human and environment. 3. Village government institutes. 4. Non-government organisations. 5. UN agencies.	1. None. 2. FAO and USAID supported the rabies vaccination. 3 and 4. None. 5. Staff shortage in animal health sector 6. None. 7. USAID.	1. Meetings, informal and formal sharing of information. 2. Electronic Integrated Disease Surveillance and Response System, Afya data, Event-based Mobile Application system and users WhatsApp groups. 3. Recommendation for awareness on OH.	1. None. 2. Zonal veterinary laboratory. 3 and 4. None.	1. The National One Health Strategic Plan, 2015–2020. 2. None. 3. One Health Coordination Desk. 4. Weak enforcement of by-laws requiring animal vaccination. Lack of enforcement of public health laws.
	Tanzania, anthrax.	1. Ministries of Livestock and Fisheries, Health, Community Development, Gender, Elderly and Children and Natural Resources and Tourism. 2. Animal, human and environment. 3. Village government institutes. 4. Non-government organisations. 5. UN agencies.	1. None. 2. FAO supported anthrax vaccination. 3 and 4. None. 5. Staff shortages in animal health sector. 6. None. 7. USAID.	1. Meetings, informal and formal sharing of information. 2. Electronic Integrated Disease Surveillance and Response System, Afya data, Event-based Mobile Application system and users WhatsApp groups. 3. Recommendation for awareness on OH.	1. District and regional hospitals and dispensary. 2. The Tanzania Veterinary Laboratories Agency. 3. Lack of reagents. 4. None.	1. The National One Health Strategic Plan, 2015–2020. 2. None. 3. One Health. Coordination Desk. 4. Lack of enforcement of public health laws.

FAO=Food and Agriculture Organization of the United Nations; ID=Intradermal; OH=One Health; OIE = World Organization for Animal Health; PAPARCON = Pan African Rabies Control Network; SwissTPH = Swiss Tropical and Public Health Institute; UN = The United Nations; USAID = United States Agency for International Development; USCDC=United States Centers for Disease Control and Prevention; WHO=World Health Organization.

# Sub-domains for each domain above are sequentially listed in this column. The serial numbers for these sub-domains correspond to the information extracted from the studies in the subsequent rows. Example: In the first row, *1. Directorate of Public Health and Preventive Medicine* is a stakeholder under the *sub-domain: 1. stakeholders from the animal health, human health and environmental sectors* under the *Domain: Stakeholders, roles and responsibilities*.

#### 3.2.2 Financial and personal resources

Partial/full national funding mechanisms were acknowledged in only 6 (21%) of the studies in five articles [60,69,71,74,77,79]. For example, a study from Indonesia reported availability of fund for avian influenza outbreaks through the national disaster management agency [69] and a study from Canada reported reinvestment of the revenue generated from licensing dog ownership to promote One Health activities for rabies and dog-bites control [77]. There were 18 (62%) studies in 10 articles that indicated funding and/or technical support from various donors and international partners [34,60,66,68,69,71,73,75,76,80]. For example, a study from Thailand reported technical and financial support from FAO, WHO, USAID and US CDC for One Health process against avian influenza [69]. Three articles referred to the sustainability of the funding mechanisms in various terms. One study explicitly described about the sustainability of the funding [74], one recommended a sustained funding [73] and the other mentioned lack of sustained funding as a challenge [34]. Nine studies (31%) in eight articles did not mention funding or the financial sustainability of the One Health implementation [67,70,72,78,79,[81], [82], [83]].

Five (17%) and 14 (48%) studies in 10 articles stated low capacity of staff and staff insufficiency respectively, as a challenge to operationalising One Health. [34,60,66,[72], [73], [74],^76^,^80^,^81^,^83^] Eleven articles described trainings in 16 (55%) studies. [34,60,[68], [69], [70], [71],^73^,^75^,^78^,^79^,^81^] Eleven of these studies revealed that some sort of training was imparted whereas five expressed the need for training.

#### 3.2.3 Communication and information

We identified 23 (79%) studies in 16 articles that referred to this domain [34,[66], [67], [68], [69], [70], [71], [72], [73], [74], [75], [76], [77],^79^,^81^,^83^]. Use of both formal and informal means of communication was highlighted in the selected articles. Meetings were means of sharing information in six of the studies described in three articles [66,68,73]. Five studies in three articles mentioned the importance of informal methods of information sharing for One Health operationalisation [66,67,73]. Electronic applications were used to share information on a real-time basis in six (21%) of the studies that were described in five articles [66,[70], [71], [72], [73]]. These applications included bespoke applications for smartphones through social platforms. For example, users WhatsApp [84] (cross-platform messaging and Voice over IP service owned by Facebook, Inc.) groups were utilised for communication between government ministries, international organisations, health institutes, and medical and veterinary officials of regions and districts for anthrax control in Tanzania [66].

Awareness about zoonotic diseases or concepts of One Health among communities and stakeholders were discussed in the studies. A total of 21 (72%) studies in 14 articles reported on awareness of either One Health approaches (27%) or zoonotic diseases (45%) [34,[66], [67], [68], [69],^71^,^72^,[74], [75], [76], [77],^79^,^81^,^83^]. From the total studies that reported on awareness, 48% mentioned lack of awareness of One Health concept and diseases among communities, 33% mentioned conduct of awareness programs on rabies and animal rights targeted for communities, school and general public and 19% mentioned plans and recommendations to conduct awareness programs on One Health concepts, dog bites and diseases among stakeholders and public.

#### 3.2.4 Technical infrastructure

Eighteen (62%) of the studies in 12 articles referred to the Technical infrastructure domain [34,66,[68], [69], [70], [71], [72], [73],^75^,[80], [81], [82]]. Ten studies (35%) in six articles provided descriptions and functions of the health system set-up in either the animal or human sectors [34,66,[68], [69], [70],^82^]. For example, district hospitals provided rabies post-exposure prophylaxis in Tanzania and livestock posts confirmed the rabies status of the dogs in human dog bite cases in Republic of Guinea. More than half of the total studies (52%) in nine articles stated the names of the laboratories and partly their set-up [34,66,68,[70], [71], [72], [73],^80^,^81^]. The types and levels of laboratories mentioned depended on the scale of One Health operationalisation and ranged from community to national levels. All the studies referring to the Technical infrastructure domain, 18 (62%) highlighted limited capacity of laboratories either in terms of logistics or human resources. For example, a study from Sri Lanka reported lack of infrastructure to collect and submit samples in wildlife for rabies control program [80]and a study from India reported lack of designated laboratory staff for diagnosis of anthrax [81].

#### 3.2.5 Governance

A total of 24 (83%) of the studies in 17 articles described the Governance domain and named national One Health plans, policies or government platforms [34,60,[66], [67], [68], [69], [70], [71], [72], [73], [74], [75], [76], [77],^79^,^82^,^83^]. For example, studies from Tanzania and Republic of Guinea [34,66] described their national One Health plans, and studies from Thailand, Nigeria, India, Côte d'Ivoire, Mali, Philippines and Indonesia [60,68,69,73,76,79] mentioned One Health platforms, while studies from Malta, Serbia, Canada and Israel [67,77,82] mentioned government bylaws and policies. From a total of 14 (48%) studies in 11 articles, that named the established platforms for One Health operationalisation [34,60,66,[68], [69], [70], [71], [72], [73],^76^,^79^], nine of the 14 platforms were established specifically for One Health operationalisation and the remaining five were established broadly for collaboration and coordination against zoonotic diseases. For example, Guinea's One Health Platform in Republic of Guinea was established specifically for the purpose of One Health coordination whereas [34], the Philippines Inter-Agency Committee on Zoonoses was established broadly for collaboration across agencies [76]. Almost half the total numbers of studies (48%) described in nine articles, related the discussion on One Health operationalisation to countries' laws and legislations [34,66,67,69,74,75,77,79,82]. Of the total four studies that reported political will, two reported its absence as a limitation and other two commended its role for the success of the One Health process.

## 4 Discussion

This is the first review to assess evidence of One Health operationalisation in zoonotic diseases prevention and control, guided by the World Bank domains.

Overall, this review found that studies have described the World Bank domains to varying degrees, with the majority of studies lacking detail on One Health operationalisation along the domains. Less than half the total studies described all the domains and none of the studies described all the sub-domains.

### 4.1 Stakeholders, roles, and responsibilities

Our review identified that the Stakeholder domain was relatively well-described among the five domains. This finding can be partially attributed to the inherent nature of any zoonotic disease control program whereby stakeholders and their roles are the drivers. The evolving concept of intervention and expansion of implementation of One Health has broadened the range of stakeholders beyond those directly involved with animal, human and environmental health [85]. This expansion was evident in the studies we reviewed which involved a range of stakeholders including non-government organisations, finance, regulatory, academic and research sectors, government administrative units, social sectors, and volunteers. A more concerning result was that the environmental sector, crucial to the control of zoonotic disease, was not mentioned in about half of the reviewed articles, which was consistent with other studies that reported under-representation of environmental sector [54,86].

International organisations are important stakeholders for One Health operationalisation. This is because, these international organisations provide financial and human resources that are interdisciplinary in nature including human, animal health, environment, and other relevant stakeholders [87].

The development and utilisation of various toolkits have proven to be useful in mapping and analysis of One Health stakeholders [88,89], and there is need for development of similar tool kit or a checklist for reporting One Health operationalisation utilising a standard set or components of One Health.

### 4.2 Financial and personal resources

Our study found that majority of the reviewed studies did not discuss this domain extensively. This could be partly attributed to technical focus and varying objectives of the reviewed studies. Nevertheless, it was clear that international organisations play an important role in providing financial support to One Health processes, especially in LMICs. Similar findings were reported in other studies [90,91]. This trend is likely to continue because of emergence and re-emergence of zoonotic diseases [92,93] and establishment of several One Health initiatives [21]. However, to sustain and expand the application of One Health to fields beyond zoonotic diseases like antimicrobial resistance, food safety and ecotoxicology, a reliable and sustained financing are required [90]. The information on financing in the literature and involvement of external funders in several One Health operationalisations makes it difficult to judge the sustainability and future of One Health operationalisation described in the selected papers. We conclude that there is need to generate more research evidence in this domain to inform on different financing models and their likely outcomes in One Health operationalisation in zoonotic diseases. This will benefit both the institutions implementing the One Health approaches and the donors in terms of ensuring sustainability of such initiatives in the future.

### 4.3 Communication and information

Sharing of surveillance data and disease information between stakeholders will enable planning and enhance response to public health events [94]. Despite diverse contexts of One Health operationalisation in the selected papers, a majority of them described Communication and information domain to a great extent demonstrating its significance in the context of zoonotic diseases prevention and control. Elements of information and communication can also be found incorporated into the evaluation frameworks for One Health [95] and a review identified communication as one of the factors that support successful One Health collaboration and response to health events [96], further demonstrating its significance. Both formal and informal communication was found to be contributing to success of One Health. There is a consensus that response to health emergencies is more effective when formal and informal relationships are established [96].

Domination by discussion on awareness in the studies mentioning this domain demonstrated its significance, supporting similar findings by other authors [[97], [98], [99]]. Knowledge and awareness are proposed as one of the themes of the updated core competencies for One Health [100,101] The fact that infectious diseases outbreaks often appear at the community level, which often lacks basic health services, warrants innovative communication approaches to collect near- to real-time information, and has proven to be successful [102]. Therefore, One Health implementations should prioritise harness of the power of electronic media at local levels and link them to global One Health networks [103] in parallel with building capacity of stakeholders to collaborate across disciplines at a global level [104].

### 4.4 Technical infrastructure

Effective laboratory systems are essential for an efficient surveillance of zoonoses [105]. Despite the development of sophisticated and highly efficient laboratory technologies worldwide, challenges remain at the local community level, where the risk of zoonotic diseases is often higher [106]. Discussions on challenges and limitations have dominated the studies that have mentioned laboratory systems in this review. While there is an abundance of literature focussing on limitations and challenges of laboratories, there is a dearth of publications that provide insights into their set-up and networking. Therefore, there is need for more literature offering lessons on laboratory systems set-up and alignment for the purpose of successful One Health processes in the context of disease surveillance and monitoring in animals, humans, and environment. Appropriately established laboratory systems can reduce expenditures and response times through One Health *via* sharing of information and physical resources [107].

Further, laboratory assessment tools, such as the Laboratory Mapping Tool [108] has been used by more than 30 countries [109] to evaluate and develop laboratory systems in animal health sectors. Similar tools such as WHO-Laboratory Assessment Tool [110] and Assessment Tools for Laboratory Services [111] are available for human health sector. Utilisation of such tools and reporting their outcomes will help in assuring quality laboratory systems are in place for successful One Health operations.

### 4.5 Governance

Governance with capacity to prepare for complex challenges and multi-sector responses are required for successful implementation of the One Health approach [112]. It also requires processes, rules, and institutions to enable policy and practice to be co-managed and co-delivered [113]. We found that national policies and One Health platforms are critical components of successful One Health processes. These improve coordination and integration of activities and programs across sectors [12]. The platforms and networks facilitate integrated engagements of multiple disciplines beyond zoonotic diseases such as antimicrobial resistance, food safety, non-communicable diseases, and climate changes [114]. Our study also found that majority of the publications emphasised the importance of the governance and mentioned the relevant policies that impact the One Health process. Although the selected literature described operationalisation of One Health within diverse government settings, the importance of governance, supporting policies and formation of appropriate One Health platforms for successful One Health processes were consistently implemented.

### 4.6 Limitations

This review was limited to the studies that described the implementation of the One Health approach in the context of the five zoonotic diseases with relevance to One Health approach globally. Therefore, we cannot confirm that findings and discussions presented in this study are generalisable to studies on all zoonotic diseases. However, the One Health approach and its principles are applicable to all zoonotic disease, including to emerging and re-emerging diseases of recent times.

The review was guided by the domains of the World Bank, aligning to the fundamental principles of the One Health approach. The sub-domains were extracted from other relevant studies and do not constitute all the components discussed in the World Bank operational framework. Therefore, the findings from this review should be applied with due consideration of these limitations.

## 5 Conclusion

Despite limiting the review to the five zoonotic diseases, this study has revealed some significant findings regarding the reporting of One Health operationalisation across the domains. Overall, the lack of evidence on operationalisation across the domains discovered by this review indicates either that such One Health operationalisation is not sufficiently prioritised and that there is no standardised framework for reporting One Health implementation across the five domains of the World Bank.

## Funding

This research is supported by an Australian Government Research Training Program (RTP) scholarship.

## Ethics approval

Not applicable.

## CRediT authorship contribution statement

**Bir Doj Rai:** Conceptualization, Data curation, Formal analysis, Investigation, Methodology, Writing – original draft, Writing – review & editing. **Gizachew A. Tessema:** Conceptualization, Data curation, Methodology, Supervision, Validation, Writing – review & editing. **Lin Fritschi:** Conceptualization, Methodology, Supervision, Validation, Writing – review & editing. **Gavin Pereira:** Conceptualization, Methodology, Supervision, Validation, Writing – review & editing.

## Declaration of competing interest

The authors declare the following financial interests/personal relationships which may be considered as potential competing interests.

The corresponding author is a recipient of the Australian Government Research Training Program (RTP) scholarship. If there are other authors, they declare that they have no known competing financial interests or personal relationships that could have appeared to influence the work reported in this paper.

## Data Availability

I have attached a supplementary file with associated information used in the manuscript.

## Appendix A Supplementary data

Supplementary file 1. Definitions of One Health by Organizations Image 1

Supplementary file 2. PRISMA-ScR Checklist Image 2

Supplementary file 3. Inclusion and exclusion criteria Image 3

Supplementary file 4. Concepts grid and search strategy Image 4

Supplementary file 5. Data Chart Image 5
