# Supplementary material for: The application of the One Health approach in the management of five major zoonotic diseases using the World Bank domains: A scoping review
Source: One Health. 2024 Feb 15;18:100695. doi: 10.1016/j.onehlt.2024.100695 (PMC11247293; doi:10.1016/j.onehlt.2024.100695)
Supplement: Supplementary file 1 — Definitions of One Health by Organizations [file mmc1.docx]

Supplementary file 1. Definitions of One Health by Organizations

| Organization | Definition |
| --- | --- |
| Centers for Disease Control and Prevention | One Health is a collaborative, multisectoral, and transdisciplinary approach- working at the local, regional, national, and global levels- with the goal of achieving optimal health outcomes recognizing the interconnection between people, animals, plants, and their shared environment.^3^ |
| World Health Organization | 'One Health' is an approach to designing and implementing programmes, policies, legislation and research in which multiple sectors communicate and work together to achieve better public health outcomes.^9^ |
| One Health Commission | One Health is the collaborative effort of multiple health science professions, together with their related disciplines and institutions – working locally, nationally, and globally – to attain optimal health for people, domestic animals, wildlife, plants, and our environment.^4, 6^ |
| One Health Global Network | Approach to improve health and well-being through the prevention of risks and the mitigation of effects of crises that originate at the interface between humans, animals, and their various environments.^4, 7^ |
| American Veterinary Medical Association | The integrative effort of multiple disciplines working locally, nationally, and globally to attain optimal health for people, animals and the environment.^5^ |
| The World Bank framework | A collaborative approach for strengthening systems to prevent, prepare, detect, respond to, and recover from primarily infectious diseases and related issues such as antimicrobial resistance that threatens human health, animal health, and environmental health collectively, using tools such as surveillance and reporting with an endpoint of improving global health security and achieving gains in development. While using infectious disease/AMR as a starting point, we recognize this definition and approach is expandable for wider scope (e.g., water and soil pollution that have animal and environment connections).^8^ |
| One Health High-Level Expert Panel | One Health is an integrated, unifying approach that aims to sustainably balance and optimize the health of people, animals, and ecosystems.  It recognizes the health of humans, domestic and wild animals, plants, and the wider environment (including ecosystems) are closely linked and inter-dependent.  The approach mobilizes multiple sectors, disciplines and communities at varying levels of society to work together to foster well-being and tackle threats to health and ecosystems, while addressing the collective need for clean water, energy and air, safe and nutritious food, taking action on climate change, and contributing to sustainable development.^2^ |
