# Supplementary material for: The application of the One Health approach in the management of five major zoonotic diseases using the World Bank domains: A scoping review
Source: One Health. 2024 Feb 15;18:100695. doi: 10.1016/j.onehlt.2024.100695 (PMC11247293; doi:10.1016/j.onehlt.2024.100695)
Supplement: Supplementary file 2 — PRISMA-ScR Checklist [file mmc2.docx]

Supplementary file 2. PRISMA-ScR Checklist

| **Section and Topic** | **Item** | **PRISMA-ScR CHECKLIST ITEM** | **Page reported on** |
| --- | --- | --- | --- |
| **TITLE** | | |  |
| Title | 1 | Identify the report as a scoping review. | 1 |
| **ABSTRACT** | | |  |
| Structured summary | 2 | Provide a structured summary including, as applicable: background, objectives, eligibility criteria, sources of evidence, charting methods, results, and conclusions that relate to the review question(s) and objective(s). | 3 (As abstract) |
| **INTRODUCTION** | | |  |
| Rationale | 3 | Describe the rationale for the review in the context of what is already known. Explain why the review question(s)/objective(s) lend themselves to a scoping review approach. | 4 |
| Objectives | 4 | Provide an explicit statement of the question(s) and objective(s) being addressed with reference to their key elements (e.g., population or participants, concepts, and context), or other relevant key elements used to conceptualize the review question(s) and/or objective(s)). | 7 |
| **METHODS** | | |  |
| Protocol and registration | 5 | Indicate if a review protocol exists, if and where it can be accessed (e.g., web address), and, if available, provide registration information including registration number. | Not registered |
| Eligibility criteria | 6 | Specify the characteristics of the sources of evidence (e.g., years considered, language, publication status) used as criteria for eligibility, and provide a rationale. | 7 |
| Information sources | 7 | Describe all information sources (e.g., databases with dates of coverage, contact with authors to identify additional sources) in the search, as well as the date the most recent search was executed | 7 |
| Search | 8 | Present the full electronic search strategy for at least one database, including any limits used, such that it could be repeated. | 7  (Details in supplementary file 4) |
| Selection of sources of evidence | 9 | State the process for selecting sources of evidence (i.e., screening, eligibility) included in the scoping review. | 7  (Details in PRISMA Flowchart-Figure 1) |
| Data charting process | 10 | Describe the methods of charting data from the included sources of evidence (e.g., piloted forms; forms that have been tested by the team before their use, whether data charting was done independently, in duplicate) and any processes for obtaining and confirming data from investigators. | 8 (Details in supplementary file 5 as data chart) |
| Data items | 11 | List and define all variables for which data were sought and any assumptions and simplifications made. | 8 |
| Critical appraisal of individual sources of evidence | 12 | If done, provide a rationale for conducting a critical appraisal of included sources of evidence; describe the methods used and how this information was used in any data synthesis (if appropriate). | Not done |
| Synthesis of results | 13 | Describe the methods of handling and summarizing the data that were charted. | 9 |
| **RESULTS** | | |  |
| Selection of sources of evidence | 14 | Give numbers of sources of evidence screened, assessed for eligibility, and included in the review, with reasons for exclusions at each stage, ideally using a flow diagram. | 9  (Details in Figure 1 as PRISMA flowchart) |
| Characteristics of sources of evidence | 15 | For each source of evidence, present characteristics for which data were charted and provide the citations. | 9 (Table 2) |
| Critical appraisal within sources of evidence | 16 | If done, present data on critical appraisal of included sources of evidence (see item 12). | No done |
| Results of individual studies | 17 | For each included source of evidence, present the relevant data that were charted that relate to the review questions and objectives. | 10 (Table 3) |
| Synthesis of results | 18 | Summarize and/or present the charting results as they relate to the review questions and objectives. | 10 (Table 3) |
| **DISCUSSION** | | |  |
| Summary of evidence | 19 | Summarize the main results (including an overview of concepts, themes, and types of evidence available), link to the review questions and objectives, and consider the relevance to key groups. | 13 |
| Limitations | 20 | Discuss the limitations of the scoping review process. | 17 |
| Conclusions | 21 | Provide a general interpretation of the results with respect to the review questions and objectives, as well as potential implications and/or next steps. | 18 |
| **FUNDUNG** | | |  |
| Funding | 22 | Describe sources of funding for the included sources of evidence, as well as sources of funding for the scoping review. Describe the role of the funders of the scoping review. | 18 |

Adapted from Tricco AC, Lillie E, Zarin W, et al. PRISMA Extension for Scoping Reviews (PRISMA-ScR): Checklist and Explanation. *Ann Intern Med*. 2018;169(7):467-473. doi:10.7326/M18-0850 ^64^
