# Supplementary material for: The application of the One Health approach in the management of five major zoonotic diseases using the World Bank domains: A scoping review
Source: One Health. 2024 Feb 15;18:100695. doi: 10.1016/j.onehlt.2024.100695 (PMC11247293; doi:10.1016/j.onehlt.2024.100695)
Supplement: Supplementary file 3 — Inclusion and exclusion criteria [file mmc3.docx]

Supplementary file 3. Inclusion and exclusion criteria

| **Inclusion** | **Exclusion** |
| --- | --- |
| - Studies reporting operationalisation of One Health in prevention and control of one of the selected zoonotic diseases; anthrax, avian influenza, brucellosis, rabies, scrub typhus. - Studies irrespective of their design. - Only primary research papers, published in peer-reviewed journals., - Articles published in English from 2004 to 2022. | - Studies or analysis not elaborating on principles of OH and not implemented to the five zoonotic disease prevention and control. - Secondary research, editorials, commentaries, erratum, pre-prints, reviews. |
