# Supplementary material for: The application of the One Health approach in the management of five major zoonotic diseases using the World Bank domains: A scoping review
Source: One Health. 2024 Feb 15;18:100695. doi: 10.1016/j.onehlt.2024.100695 (PMC11247293; doi:10.1016/j.onehlt.2024.100695)
Supplement: Supplementary file 4 — Concepts grid and search strategy [file mmc4.docx]

Supplementary file 4. Concepts grid and search strategy

| **Concept 1** |  | **Concept 2** | |  | **Concept 3** |  | **Concept 4** |  | **Concept 5** |  | **Concept 6** |
| --- | --- | --- | --- | --- | --- | --- | --- | --- | --- | --- | --- |
| One Health | AND | Rabies | | OR | Anthrax | OR | Scrub Typhus | OR | Brucellosis | OR | Avian Influenza |
| **Key terms and synonyms** | | | | | | | | | | | |
| Rabies | | | hydrophobia or lyssa* or "canine madness" or rabid | | | | | | | | |
| Anthrax | | | "Siberian plague" or "cumberland disease" or "splenic fever" or charbon or "malignant edema" or "woolsorter* disease" or "ragpicker* disease" or "black baine" or "malignant pustule" | | | | | | | | |
| Scrub Typhus | | | "bush typhus" or "tsutsugamushi disease" | | | | | | | | |
| Brucellosis | | | "bang's disease" or "malta fever" or "mediterranean fever" or "undulant fever" or "crimean fever" | | | | | | | | |
| Avian Influenza | | | H5N1 | | | | | | | | |
| **Search Syntax** | | | | | | | | | | | |
| "One Health" AND (Rabies or hydrophobia or lyssa* OR "canine madness" or rabid OR anthrax OR "siberean plague" OR "cumberland disease" OR "splenic fever" OR charbon OR "malignant edema" OR "woolsorter* disease" OR "ragpicker* disease" OR "black baine" OR "malignant pustule" OR brucell* OR "bang's disease" OR "malta fever" OR "mediterranean fever" OR "undulant fever" OR "crimean fever" OR "avian influenza" or H5N1 OR "scrub typhus" or "bush typhus" or "tsutsugamushi disease")  Filters  Period- 2004 – 2022  Language- English | | | | | | | | | | | |
