# Supplementary material for: The application of the One Health approach in the management of five major zoonotic diseases using the World Bank domains: A scoping review
Source: One Health. 2024 Feb 15;18:100695. doi: 10.1016/j.onehlt.2024.100695 (PMC11247293; doi:10.1016/j.onehlt.2024.100695)
Supplement: Supplementary file 5 — Data Chart [file mmc5.docx]

Supplementary file 5. Data Chart

| 1. Author(s) |
| --- |
| 1. Year |
| 1. Origin/country of study |
| 1. Aim/purpose |
| 1. Zoonoses against which OH was operationalised |
| 1. Domains (5)   Sub-domains (23) |
